# Climate change impedes plant immunity mechanisms

**DOI:** 10.3389/fpls.2022.1032820

**Published:** 2022-11-29

**Authors:** Seungmin Son, Sang Ryeol Park

**Affiliations:** National Institute of Agricultural Sciences, Rural Development Administration, Jeonju, South Korea

**Keywords:** carbon dioxide, climate change, crop nutritional security, humidity, pathogen, plant immunity, temperature

## Abstract

Rapid climate change caused by human activity is threatening global crop production and food security worldwide. In particular, the emergence of new infectious plant pathogens and the geographical expansion of plant disease incidence result in serious yield losses of major crops annually. Since climate change has accelerated recently and is expected to worsen in the future, we have reached an inflection point where comprehensive preparations to cope with the upcoming crisis can no longer be delayed. Development of new plant breeding technologies including site-directed nucleases offers the opportunity to mitigate the effects of the changing climate. Therefore, understanding the effects of climate change on plant innate immunity and identification of elite genes conferring disease resistance are crucial for the engineering of new crop cultivars and plant improvement strategies. Here, we summarize and discuss the effects of major environmental factors such as temperature, humidity, and carbon dioxide concentration on plant immunity systems. This review provides a strategy for securing crop-based nutrition against severe pathogen attacks in the era of climate change.

## Introduction

Climate change is a major factor in determining where humans can live on the planet under tolerable and safe conditions (Timmermann et al., 2022). Global warming due to environmental destruction and excessive burning of fossil fuels is creating adverse conditions for the continued survival of many plant and animal species and the wellness of the human population (Román-Palacios and Wiens, 2020). The crops that have made human settlement possible since the dawn of agriculture by providing a stable source of dietary calories are now suffering from the effects of climate change (Challinor et al., 2014; Rising and Devineni, 2020). Biotic stress factors such as pathogens and insect pests reduce crop yield and quality in agricultural settings (Savary et al., 2019; Savary and Willocquet, 2020). Indeed, damage to major crop yields is estimated to reach up to 40% globally (Oerke, 2006; Savary et al., 2012). In warmer and wetter environments more amenable to pathogen growth and spread, the damage they cause can be even more devastating (Velasquez et al., 2018). For example, bacterial blight caused by *Xanthomonas oryzae* pv. *oryzae* (*Xoo*) can decrease yield in rice (*Oryza sativa*) by up to 80% (Srinivasan and Gnanamanickam, 2005). Wheat blast caused by the fungus *Magnaporthe oryzae Triticum* can infect wheat (*Triticum aestivum*) and completely eradicate fields (Islam et al., 2020), as can banded leaf and sheath blight caused by *Rhizoctonia solani* in maize (*Zea mays*) (Haque et al., 2022). Moreover, the emergence of new pathogenic strains and the expansion of their effective damage zones due to climate change are two of the most serious threats to crop production and food security (Chaloner et al., 2021). Therefore, efficient strategies are urgently needed to reduce the impact of pathogens on crop growth and yield.

According to the disease triangle model, three factors are required for disease development: a susceptible host, a virulent pathogen, and a favorable environment (Scholthof, 2007). Of these, only plant-based strategies are available to affect one side of the triangle with current technologies. Indeed, the development of new crop cultivars conferring innate immunity will be essential for conservation of food resources. Plant breeding has traditionally been performed through laborious and time-consuming genetic crosses to introduce superior alleles into a given background (Lusser et al., 2012). However, biotechnological innovations now offer eight new plant breeding technologies (NPBTs): site-directed nucleases (SDNs), oligonucleotide-directed mutagenesis, cisgenesis and intragenesis, RNA-dependent DNA methylation, grafting, reverse breeding, Agrobacterium-mediated infiltration, and synthetic genomics (Lusser et al., 2011). Among them, SDNs are the most widely used NPBT for a broad range of crops. In particular, development of the clustered regularly interspaced short palindromic repeats (CRISPR)/CRISPR-associated nuclease 9 (Cas9) system has ushered in a new era of crop improvement (Son and Park, 2022). Therefore, understanding the molecular mechanisms and identifying novel genes conferring desired traits are essential for their targeting by NPBTs in plant breeding.

Plants have evolved varied stress responses and defense mechanisms to overcome adverse environmental conditions, about which we have gained a wealth of knowledge thanks to the efforts of countless scientists. Nevertheless, how climate change affects the molecular mechanisms related to plant immunity against pathogens is largely unknown. Luckily, this knowledge gap is beginning to be filled. In this review, we give an overview and discuss the negative effects of temperature, humidity, and carbon dioxide (CO_2_) concentration on plant defense mechanisms to better understand how to design mitigation strategies.

## Plant immunity system and defense signaling

Plants employ two important immune systems known as pathogen-associated molecular pattern (PAMP)-triggered immunity (PTI) and effector-triggered immunity (ETI) to perceive and respond to pathogen attacks (Thomma et al., 2011). PTI is activated mainly by plasma membrane–localized extracellular pattern recognition receptors (PRRs) that can recognize conserved PAMPs (Monaghan and Zipfel, 2012). For example, recognition of the 22–amino acid region of bacterial flagellin (flg22) by the leucine-rich repeat receptor kinase (LRR-RK) FLAGELLIN SENSING 2 (FLS2) at the plasma membrane leads to formation of a heteromer between FLS2 and BRASSINOSTEROID INSENSITIVE-ASSOCIATED KINASE 1 (BAK1), a member of the LRR receptor-like kinase (LRR-RLK) and also known as SOMATIC EMBRYOGENESIS RECEPTOR-LIKE KINASE 3 (SERK3) (Chinchilla et al., 2007). The FLS2/BAK1 complex phosphorylates the receptor-like cytoplasmic kinase BOTRYTIS-INDUCED KINASE 1 (BIK1) and mitogen-activated protein kinase (MAPK) cascade to activate the downstream signaling pathway, resulting in expression of PTI-related genes (Wang et al., 2020b). Similarly, perception of a highly conserved epitope of bacterial translation elongation factor Tu (EF-Tu) by the LRR-RK EF-Tu RECEPTOR (EFR) also results in PTI activation through heteromerization with BAK1 and phosphorylation of BIK1 (Lal et al., 2018). Moreover, the recognition of plant-derived damage-associated molecular patterns (DAMPs) and phytocytokines by LRR-RKs/RLKs is important for PTI (Hou et al., 2021; Tanaka and Heil, 2021). PTI acts as a basal defense mechanism against various types of pathogens through defense responses that include the induction of defense gene expression, reactive oxygen species (ROS) production, callose deposition, and accumulation of antimicrobial secondary metabolites (Naveed et al., 2020).

ETI is triggered following the recognition by intracellular receptor resistance (R) proteins of specific pathogen effectors that can neutralize the plant immune system in the cytoplasm (Chisholm et al., 2006; Jones and Dangl, 2006). ETI activates a prolonged and robust resistance response and rapid localized programmed cell death known as the hypersensitive response (HR) (Coll et al., 2011). Most R proteins are nucleotide-binding leucine-rich repeat proteins (NLRs) that can be classified into three groups based on their N terminus domain: Toll/interleukin-1 receptor (TIR), coiled-coil (CC), and RESISTANCE TO POWDERY MILDEW 8 (RPW8)-type CC (CC_R_) domain (Monteiro and Nishimura, 2018). The ETI signal triggered by TIR-NLRs (TNLs) relies on the three acyl hydrolases ENHANCED DISEASE SUSCEPTIBILITY 1 (EDS1), PHYTOALEXIN DEFICIENT 4 (PAD4), and SENESCENCE-ASSOCIATED GENE 101 (SAG101) (Wiermer et al., 2005). EDS1 interacts directly with PAD4 or SAG101 to form exclusive heterodimers, each with distinct functions in immunity (Wagner et al., 2013; Lapin et al., 2020). It was recently revealed that helper CC_R_-NLRs such as ACTIVATED DISEASE RESISTANCE 1 (ADR1) and N REQUIREMENT GENE 1 (NRG1) are required for the activation of the EDS1 complex and TNL defense signaling (i.e., EDS1–PAD4–ADR1 and EDS1–SAG101–NRG1) (Pruitt et al., 2021; Sun et al., 2021). The EDS1 pathway is involved not only in ETI but also in basal immunity and promotes salicylic acid (SA) biosynthesis and signaling (Cui et al., 2017). Therefore, EDS1 signaling plays a critical role in SA-dependent and -independent resistance. For CC-NLRs (CNLs), the plasma membrane-localized integrin-like protein NON-RACE SPECIFIC DISEASE RESISTANCE 1 (NDR1) appears to function downstream of CNLs, although several do not require NDR1 to activate ETI (van Wersch et al., 2020). Since NDR1 acts upstream of SA biosynthesis and signaling, it is also involved in SA-dependent resistance (Shapiro and Zhang, 2001).

Another plant immune response is referred to as quantitative disease resistance (QDR), which is characterized by a continuous distribution of resistance phenotypes—from highly sensitive to highly resistant—within a population (Poland et al., 2009). QDR is typically partial resistance conferred by multiple small-effect loci, while qualitative disease resistance, also referred as ETI, is complete resistance conferred by a single large-effect gene (French et al., 2016). Since multiple genes are involved in QDR, it is important in the context of the evolutionary pressure imposed by pathogens and confers broad-spectrum resistance to a wide range of pathogens including biotrophic and necrotrophic pathogens (Anderson et al., 2010; French et al., 2016). Most loci identified as quantitative trait loci for QDR are associated with the biosynthesis of the cell wall and defense compounds, thus extending beyond simple pathogen perception (Corwin and Kliebenstein, 2017).

Phytohormones participate in and control PTI and ETI. In particular, the three phytohormones SA, jasmonic acid (JA), and ethylene (ET) play critical roles in plant immunity. SA contributes significantly to innate immunity against biotrophic pathogens by evoking local and systemic resistance, whereas JA/ET play critical roles in plant resistance to necrotrophic pathogens (Glazebrook, 2005; Li et al., 2019). The SA and JA/ET defense signals can be antagonistic or synergistic (Tsuda and Katagiri, 2010). Abscisic acid (ABA) is also important for innate immunity. ABA interacts with various phytohormones during defense responses (Lee and Luan, 2012; Pieterse et al., 2012). For example, ABA suppresses SA-dependent immunity, leading to greater susceptibility against various pathogens (Berens et al., 2019). However, ABA can also increase plant disease resistance due to closure of stomata which constitutes one of the main entry routes for pathogens (Ton Mauch‐Mani, 2004; Melotto et al., 2006; Flors et al., 2008). In response to the stimulus, ABA is primarily biosynthesized in vascular tissues and accumulates in guard cells through ABA transporters (e.g., ATP-binding cassette transporter G [ABCG]) (Merilo et al., 2015). In guard cells, ABA binds to its cognate receptor from the pyrabactin resistance 1/pyrabactin resistance 1-like/regulatory components of ABA receptors (PYR/PYL/RCAR) family, leading to the inactivation of type 2C protein phosphatases (PP2Cs). The alleviation of PP2C-mediated repression of SUCROSE NON-FERMENTING 1 (SNF1)-related protein kinase 2s (SnRK2s) results in activation of the downstream ABA signaling cascade (Hsu et al., 2021). For example, the PP2Cs ABA INSENSITIVE 1 (ABI1) and ABI2 inactivate OPEN STOMATA 1 (OST1), also known as SnRK2.6, thus preventing the phosphorylation of SLOW ANION CHANNEL 1 (SLAC1), which releases anions for stomatal closure. However, perception of flg22 by PRRs increases ABA levels in guard cells to inactivate ABIs, and it results in rapid stomatal closure through the activation of the OST1/SnRK2.6–SLAC1 module (Guzel Deger et al., 2015). Therefore, ABA promotes stomatal closure and prevents pathogen entry into the host plant.

ROS signaling is also important for plant immunity. ROS are highly oxidative agents, but they also act as signaling molecules that regulate biotic stress responses (e.g., systemic acquired resistance [SAR] and cell death) (Waszczak et al., 2018). ROS are generated *via* metabolic and stress signaling pathways. Metabolic ROS are produced in several intracellular compartments (e.g., chloroplast, mitochondria, peroxisomes, and apoplast) during photosynthesis and photorespiration, while signaling ROS are produced mainly by plant NADPH oxidases, mostly from members of the plasma membrane–localized respiratory burst oxidase homolog (RBOH) family (Kangasjärvi et al., 2012; Chapman et al., 2019). Pathogen recognition is accompanied by ROS production through both the metabolic and stress signaling pathways. Recognition of PAMPs by PRRs induces an initial oxidative burst that activates plant basal defenses within the infected cells; effector perception by R proteins then promotes a second oxidative burst that results in HR (Nanda et al., 2010; Torres, 2010). Therefore, ROS play a key role linking pathogen perception and plant defense responses.

However, these various plant defense systems may be adversely affected significantly by climate change, as discussed below.

## The effects of temperature on PTI

Environmental factors influence not only pathogenicity but also plant disease resistance (Elad and Pertot, 2014). Temperature is perhaps the most studied climate factor modulating plant–pathogen interactions. Higher average temperatures brought upon by climate change can increase the pathogenicity of phytopathogens by raising their virulence, active geographical regions, fitness, reproduction period/rate, and epidemic risks (Agrios, 2005; Deutsch et al., 2008; Caffarra et al., 2012; Vaumourin and Laine, 2018). Temperature is also one of the most important environmental factors that shapes plant immunity against bacteria, fungi, viruses, and insects (Garrett et al., 2006). Since different host–pathogen interactions behave differently over different temperature ranges, higher temperatures will sometimes work in favor of plant immunity. In many cases though, higher temperature will benefit the pathogen to the detriment of the host (Desaint et al., 2021).

In Arabidopsis (*Arabidopsis thaliana*), higher temperature increases early PTI signaling (through BIK1 and MAPKs) and decreases the occupancy of nucleosomes containing the histone variant H2A.Z, which modulates the plant transcriptome in response to changes in temperature (Kumar and Wigge, 2010; Cheng et al., 2013). Moderately high temperatures (23°C–32°C) will therefore activate PTI-dependent gene expression at the expense of ETI (Cheng et al., 2013). Cysteine-rich receptor-like kinases (CRKs) are one of the largest RLK subfamilies that recognizes pathogens and activates downstream signaling cascades. Recently, Wang et al. identified a CRK from wheat cultivar ‘XY 6’ conferring high-temperature seedling-plant resistance (Wang et al., 2021). The expression level of this gene, *TaCRK10*, was induced significantly by infection with the fungal pathogen *Puccinia striiformis f.* sp. *tritici* causing strip rust at high temperature. TaCRK10 was shown to directly phosphorylate histone H2A in wheat (TaH2A.1) and activate the SA signaling pathway, resulting in enhanced high-temperature seedling-plant resistance to *P. striiformis f.* sp. *tritici* (Wang et al., 2021). However, several studies have also indicated that PTI can be compromised at high temperature upon inhibition of flg22- and SA-induced defense responses (Rasmussen et al., 2013; Huot et al., 2017; Janda et al., 2019). Therefore, further studies are needed to understand the effect of temperature on PTI in detail.

## The effects of temperature on ETI and SA-dependent immunity

Unlike PTI, much work has shown that high temperature decreases immunity evoked by ETI and QDR; this topic was well covered by a previous review (Desaint et al., 2021). Therefore, we focus here on recent important discoveries that illustrate how plant defense mechanisms are affected by high temperature.

Disruptions of NLR- and SA-mediated defense signaling by high temperature are thought to be the main reason behind diminished plant innate immunity against pathogens under these conditions. In Arabidopsis, the photoreceptor phytochrome B (phyB) also acts as a thermosensor, whereby far-red light and high temperatures lead to its inactivation (Jung et al., 2016; Legris et al., 2016). DE-ETIOLATED 1 (DET1) and CONSTITUTIVELY PHOTOMORPHOGENIC 1 (COP1), which are two key negative regulators of photomorphogenesis, promote the transcription of *PHYTOCHROME INTERACTION FACTOR 4* (*PIF4*), which encodes a basic-helix-loop-helix (bHLH) transcription factor acting as a positive regulator of growth and negative regulator of immunity (Gangappa et al., 2017; Gangappa and Kumar, 2018). phyB inhibits COP1 and PIF4 to modulate the trade-off between growth and defense. However, inactivation of phyB by high temperature results in the activation of the DET1/COP1–PIF4 module. As a result, PIF4 represses the expression of *SUPPRESSOR OF NPR1-1, CONSTITUTIVE 1* (*SNC1*), which encodes a TNL initiating ETI through the EDS1-PAD4 signaling pathway at high temperature (Gangappa et al., 2017). Since SNC1 and EDS1 play a critical role in plant defense responses such as SA biosynthesis (Zhang et al., 2003; García et al., 2010), the inhibition of *SNC1* expression at high temperature also significantly hinders SA-dependent resistance. Moreover, the SUMO E3 ligase SIZ1 (SAP and MIZ1 DOMAIN-CONTAINING LIGASE1) not only inhibits SNC1-dependent immune response but also enhances COP1 function at elevated ambient temperature (Hammoudi et al., 2018). Therefore, the activation of negative regulators (e.g., PIF4 and SIZ1) of SNC1 lead to impaired ETI and SA-dependent immunity. Recently, the transcription factor bHLH059 was identified as a temperature-responsive regulator for SA-dependent immunity acting independently of PIF4 (Bruessow et al., 2021). Relative *bHLH059* transcript level increased at 22°C compared to 16°C in Arabidopsis ecotype Columbia (Col-0). Total SA contents and resistance to *Pseudomonas syringae* pv. *tomato* (*Pst*) DC3000 decreased at 22°C relative to 16°C in Col-0, but remained similar in the *bhlh59* mutant regardless of ambient temperature. Moreover, bHLH059 has the potential to be a negative regulator involved in a defense hub associated with multiple NLRs (Mukhtar et al., 2011), hinting at a new mechanism for the temperature-mediated vulnerability of plant immune responses that should be explored in more detail.

SA is major defense phytohormone involved in PTI, ETI, and SAR; importantly, SA-dependent immunity is repressed by high temperature (Velásquez et al., 2018; Zhang and Li, 2019; Castroverde and Dina, 2021), whereas JA/ET defense signaling are enhanced under elevated temperature (Havko et al., 2020; Huang et al., 2021a). Therefore, any susceptibility to temperature in the context of plant disease resistance is mainly associated with SA signaling. SA is synthesized through the isochorismate synthase (ICS) and phenylalanine ammonia-lyase (PAL) pathways in plants (Lefevere et al., 2020). Especially, pathogen-induced SA production takes place in chloroplasts, from which it is exported to the cytoplasm *via* the SA transporter EDS5 (Serrano et al., 2013). SA activates NONEXPRESSOR OF PATHOGENESIS-RELATED GENES 1 (NPR1), the master regulator of SA signaling in the cytosol, resulting in the nuclear translocation of NPR1 to induce the expression of *pathogenesis-related* (*PR*) genes conferring disease resistance and SAR (Backer et al., 2019). Moreover, although ETI activates SA signaling, SA and NPR1 repress ETI-induced cell death *via* the formation of SA-induced NPR1 condensates to promote the degradation of proteins (e.g., NLRs, EDS1, WRKY54, and WRKY70) involved in HR (Zavaliev et al., 2020).

Huot et al. showed that inhibition of ICS1, which is also called SALICYLIC ACID-INDUCTION DEFICIENT 2 (SID2), under high-temperature conditions raised the susceptibility of Arabidopsis to *Pst* DC3000 due to the loss of SA biosynthesis and SA defense signaling (Huot et al., 2017). Furthermore, Arabidopsis disease resistance to *Pst* DC3000 increased at low temperature due to greater SA signaling that can itself be repressed by JA/ET defense signals (Li et al., 2020). However, the molecular mechanisms determining the temperature sensitivity of the SA defense signaling pathway were unknown.

**Figure 1:**
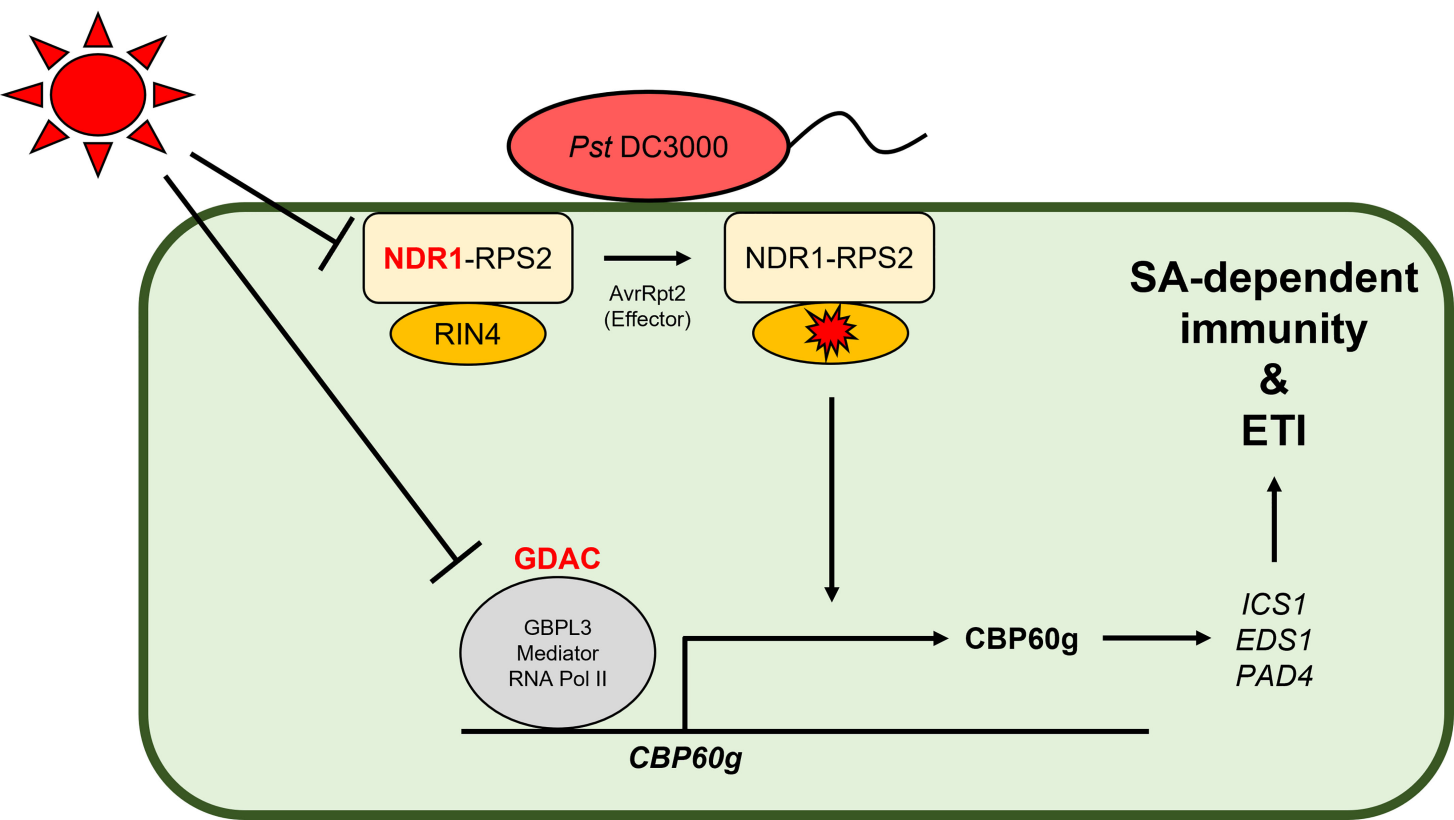
Molecular mechanisms demonstrating the negative effect of high temperature on SA-dependent immunity and ETI. In Arabidopsis, the induction of *CALMODULIN BINDING PROTEIN* 60g (CBP60g) and *NON-RACE SPECIFIC DISEASE RESISTANCE 1 (NDR1)* is necessary for innate immunity against *Pst* DC3000. However, under high temperature, the formation of guanylate binding protein-like GTPase (GBPL) defense-activated condensate (GDAC), consisting of GBPL3, Mediator, and RNA polymerase II, at the CBP60g loci (Kim et al., 2022) and the expression of *NDR1* which can increase the transcript levels of *RESISTANCE TO P. SYRINGAE 2 (RPS2)* and SA-associated genes (Samaradivakara et al., 2022) are repressed significantly, resulting in temperature vulnerability of SA-dependent immunity and ETI.

Recently, two groups demonstrated different mechanisms by which the SA-mediated immune system is modulated under high temperature (**Figure 1**). Kim et al. showed that the expression of SA response genes is decreased under elevated temperature in various dicot (e.g., Arabidopsis, rapeseed [*Brassica napus*], tobacco [*Nicotiana tabacum*], and tomato [*Solanum lycopersicum*]) and monocot (rice) plants, with the downregulation of *CALMODULIN BINDING PROTEIN 60g* (*CBP60g*) being key for the temperature vulnerability of SA defense signaling in Arabidopsis (Kim et al., 2022). GUANYLATE BINDING PROTEIN-LIKE GTPase 3 (GBPL3) binds to the promoter region of genes involved in the plant immune system and recruits the Mediator complex and RNA polymerase II to form GBPL defense–activated condensates (GDACs) (Huang et al., 2021b). The recruitment of GBPL3 and the formation of the GDAC at the *CBP60g* and *SYSTEMIC ACQUIRED RESISTANCE DEFICIENT 1* (*SARD1*) loci, which have partially redundant functions, were necessary for their transcription, and these were attenuated by heat stress (Kim et al., 2022). Therefore, the expression of various genes (e.g., ICS1, EDS1, and PAD4) that would normally induce TNL-mediated ETI and SA biosynthesis downstream of CBP60g and SARD1 was suppressed under elevated temperature. However, and surprisingly, optimized *CBP60g* expression was sufficient to restore SA accumulation and plant immune responses at high temperature without growth or developmental penalty (Kim et al., 2022). Another group unraveled the molecular mechanism explaining the temperature vulnerability of CNLs and SA defense signaling in Arabidopsis (Samaradivakara et al., 2022). *RESISTANCE TO P. SYRINGAE PV. MACULICOLA 1* (*RPM1*) and *RESISTANCE TO P*. *SYRINGAE 2* (*RPS2*) encode two CNLs that recognize type III bacterial effectors indirectly through RPM1-INTERACTING PROTEIN 4 (RIN4) (Mackey et al., 2002; Mackey et al., 2003). *P*. *syringae* bacterial effectors such as AvrRpm1 and AvrB activate RPM1-mediated ETI through hyperphosphorylation of RIN4, while AvrRpt2 activates RPS2-mediated ETI *via* the degradation of RIN4 (Axtell and Staskawicz, 2003; Zhao et al., 2021). Plasma membrane–localized NDR1 interacts with RIN4 and is required for the activation of RPS2-based ETI in response to AvrRpt2 (Belkhadir et al., 2004; Coppinger et al., 2004; Day et al., 2006). Samaradivakara et al. showed that overexpression of *NDR1* rescues the transcript levels of *RPS2* and SA-associated genes including those of *ICS1* and *CBP60g*, which are repressed by high temperature, thus resulting in enhanced resistance to *Pst* DC3000 by maintaining ETI and SA defense signaling under elevated temperature (29°C) (Samaradivakara et al., 2022). In wheat, CNLs such as TaRPM1 and TaRPS2 also positively regulate disease resistance to *P. striiformis* f. sp. *tritici* at high temperature through the SA signaling pathway (Wang et al., 2020a; Hu et al., 2021a).

## The effects of temperature on cytokinin-dependent immunity

A recent study revealed that the phytohormone cytokinin (CK) also plays an important role in plant immunity at high temperatures (Yang et al., 2022). The trade-off between growth and defense modulated by CK can result in opposite effects on plant–pathogen interactions (Choi et al., 2011). Exogenous and endogenous CK both enhance plant resistance against biotrophic pathogens through SA-dependent and -independent immune responses, therefore exerting a potentiation (or priming) defense response activated upon pathogen attack (Conrath et al., 2015; Albrecht and Argueso, 2017). Although CK displays a synergistic effect with SA, increased SA levels can inhibit CK signaling *via* a negative feedback (Argueso et al., 2012). In addition, high concentrations of CK enhance disease resistance against biotrophic oomycetes in Arabidopsis, while low concentrations raise susceptibility (Argueso et al., 2012). CK can also increase susceptibility to pathogens not only by inhibiting the plant immune system (i.e., PTI and ROS) but also by establishing source-sink relationships (Albrecht and Argueso, 2017; McIntyre et al., 2021). In pepper (*Capsicum annuum*), Yang et al. showed that infection with *Ralstonia solanacearum*, a hemibiotrophic pathogen causing bacterial wilt, activates SA signaling at an early stage and JA signaling at a later stage in roots at ambient temperature, but these responses are both impaired at high temperature (Yang et al., 2022). Instead, *isopentenyltransferase* (*IPT*) genes, including *CaIPT5*, encoding a critical enzyme in cytokinin biosynthesis, were upregulated by *R*. *solanacearum* infection under high temperature. Surprisingly, exogenous treatment with *trans*-zeatin (tZ), the bioactive CK, significantly enhanced disease resistance to *R*. *solanacearum* in pepper, tomato, and tobacco (*Nicotiana benthamiana*) under high temperature, while SA and JA did not (Yang et al., 2022). Moreover, the authors suggested that CK triggers chromatin remodeling, resulting in the upregulation of genes encoding glutathione *S*‐transferase (e.g., *CaPRP1* and *CaMgst3*) and downregulation of genes involved in SA and JA signaling (e.g., *CaSTH2* and *CaDEF1* (Yang et al., 2022).

## The effects of temperature on calcium ion–dependent immunity

Recently, the molecular mechanisms by which high temperature affects the calcium ion (Ca^2+^)–mediated immune system have also been reported. Ca^2+^ is an important second messenger modulating various signaling pathways, including the plant immune response (Yang and Poovaiah, 2003). Biotic/abiotic stresses increase Ca^2+^ levels in plant cells; Ca^2+^ then binds to calcium-binding proteins (CBPs) and Ca^2+^ sensors (e.g., calmodulin [CaM], calmodulin-like proteins [CMLs], calcineurin B-like proteins [CBLs], and calcium-dependent protein kinases CDPKs]) (Bose et al., 2011). The Ca^2+^/CBP complex activates Ca^2+^ signaling by regulating the activity of signaling components such as kinases and transcription factors (Iqbal et al., 2020; Junho et al., 2020; Ma et al., 2020). Arabidopsis SIGNAL RESPONSIVE 1 (AtSR1), also known as CALMODULIN-BINDING TRANSCRIPTION ACTIVATOR 3 (CAMTA3), plays a central role in Ca^2+^ signaling–mediated immunity (Yuan et al., 2021a). AtSR1 acts as a negative regulator of the plant immune response by decreasing the expression of genes involved in ETI and/or SA signaling (e.g., *EDS1*, *NDR1*, *CBP60g*, *SARD1*, and *NPR1*) directly or indirectly (Du et al., 2009; Nie et al., 2012; Sun et al., 2020; Yuan et al., 2021b). Recently, Yuan and Poovaiah showed that the Ca^2+^ influx induced by *Pst* DC3000 is blocked in Arabidopsis at high temperature (30°C) compared to ambient temperature (18°C). In addition, the susceptibility to *Pst* DC3000 was reduced in the *atsr1* mutant plant compared to the wild type at both 18°C and 30°C (Yuan and Poovaiah, 2022). Moreover, the authors suggested that AtSR1 increases plant vulnerability to temperature by acting on stomatal and apoplastic immunity in an SA-dependent manner. In pepper, the expression of the WRKY transcription factor gene *CaWRKY40* is induced by *Ralstonia solanacearum* infection, high temperature, and major defense phytohormones (e.g., SA, JA, and ET), and CaWRKY40 enhances both *R*. *solanacearum* resistance and heat tolerance (DANG et al., 2013). CaWRKY40 forms positive feedback loops with CaWRKY6, BASIC LEUCINE ZIPPER 63 (CabZIP63), and CaCDPK15, all positive regulators of resistance against *R*. *solanacearum* and/or heat stress tolerance (Cai et al., 2015; Shen et al., 2016a; Shen et al., 2016b). Recently, two signaling components controlled by CaWRKY40 were identified as positive and negative regulators of *R*. *solanacearum* resistance, respectively. CaCBL1 contributes to disease resistance against *R*. *solanacearum* at high temperature and participates in the positive feedback loop with CaWRKY40 (Shen et al., 2020). However, pepper MILDEW-RESISTANCE LOCUS O5 (CaMLO5) has the opposite function in plant immunity and heat resistance (Yang et al., 2020). CaWRKY40 induces the expression of *CaMLO5* at high temperature, while CaWRKY40 represses it after *R*. *solanacearum* inoculation. CaMLO5 increases tolerance to heat stress but reduces the plant immune response against *R*. *solanacearum*. Moreover, the NAM/ATAF/CUC (NAC) transcription factor CaNAC2c was recently identified as being involved in temperature-responsive immunity (Cai et al., 2021). Expression of *CaNAC2c* was induced by both high temperature and *R*. *solanacearum* inoculation, resulting in positive effects on both thermotolerance and resistance against *R*. *solanacearum* but negative effects on pepper growth. CaNAC2c modulated the thermotolerance/immunity trade-off through differential and context-specific interactions with HEAT SHOCK PROTEIN 70 (CaHSP70) and CaNAC029. However, CaNAC2c/CaNAC029-mediated *R*. *solanacearum* resistance was impaired by ABA at high temperature, suggesting that the observed thermotolerance/immunity trade-off might be modulated by an antagonistic interaction between ABA and JA signaling (Cai et al., 2021).

## The effects of humidity on stomatal immunity

Along with temperature, humidity is an influential environmental factor during plant–pathogen interactions. In general, high humidity conditions (e.g., rainfall, high atmospheric humidity, and high soil moisture) are favorable for plant infections not only by phyllosphere pathogens but also by rhizosphere pathogens. Indeed, high humidity increases the incidence of bacterial disease and the potential threat to yield in various crops (Xin et al., 2016). In fact, humidity can be more important than temperature in predicting fungal disease outbreaks (Romero et al., 2021). Since air can maintain more water vapor at high temperature, climate change is frequently accompanied by high humidity. Therefore, understanding the effect of humidity on plant immune mechanisms will be important for ensuring food security.

**Figure 2:**
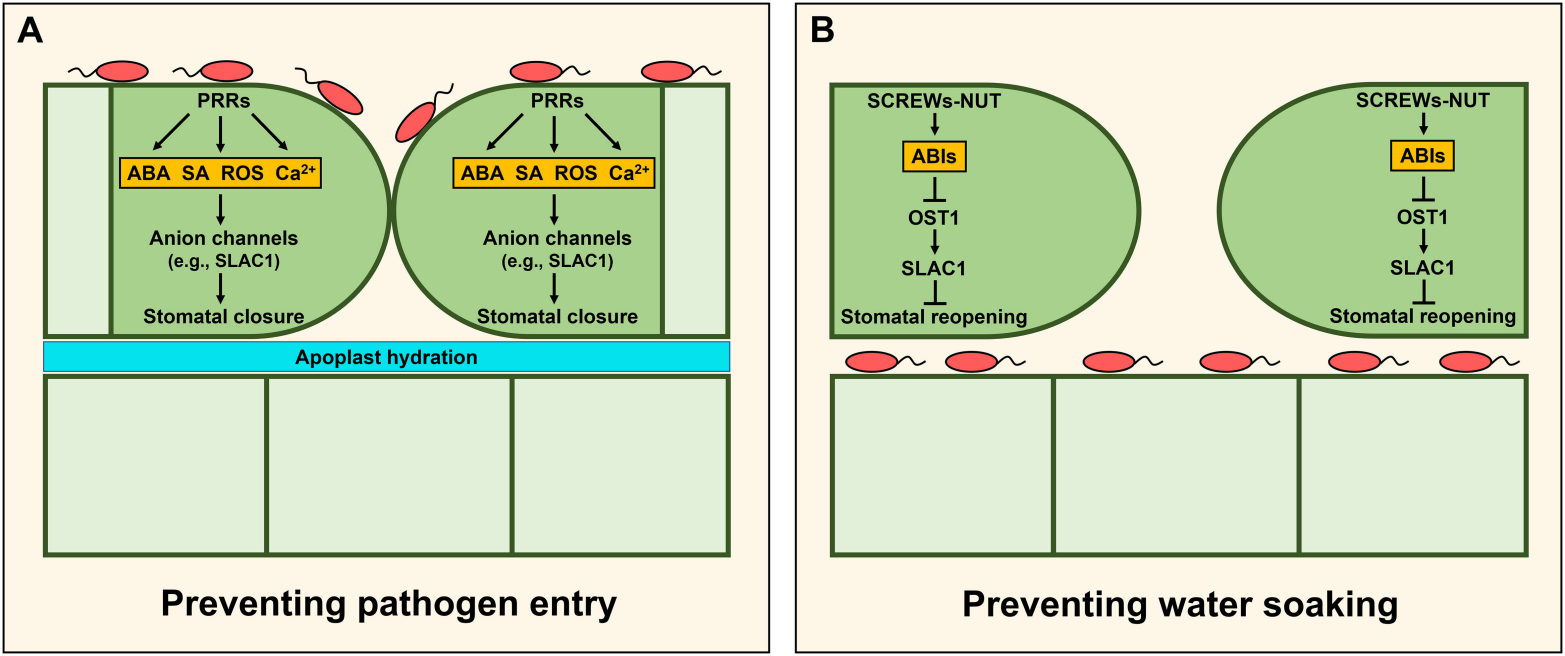
Stomatal immunity restricting pathogen entry or water soaking. **(A)** Pattern recognition receptors (PRRs)-triggered stomatal immunity. Recognition of pathogen-associated molecular patterns (PAMPs) by PRRs in guard cells promotes stomatal closure to prevent pathogen entry through activation of various signaling pathways such as ABA, SA, ROS, and Ca^2+^ (Arnaud and Hwang, 2015; Murata et al., 2015). **(B)** Stomatal immunity preventing water soaking. After pathogens invade internal plant tissues, stomatal closure can confer apoplast hydration inducing pathogen colonization. To prevent it, the secreted peptides SMALL PHYTOCYTOKINES REGULATING DEFENSE AND WATER LOSS (SCREWs) and the cognate receptor kinase PLANT SCREW UNRESPONSIVE RECEPTOR (NUT) are induced in Arabidopsis. Recognition of SCREWs by NUT increases the activity of the protein phosphatases type 2C (PP2Cs) such as ABA INSENSITIVE 1 (ABI1) and ABI2, and it results in stomatal reopening through inhibition of OST1/SnRK2.6-SLAC1 module (Liu et al., 2022b).

By far, the main target of humidity affecting plant immunity is associated with stomatal control. Stomata consist of two guard cells that play a central role in modulating water transpiration and gas exchange between the plant and the atmosphere to balance the needs of photosynthesis while minimizing drought stress. Therefore, stomatal movements are tightly regulated in response to various environmental stimuli (e.g., humidity and CO_2_) (Driesen et al., 2020). However, stomata also offer convenient portals through which pathogens can penetrate inner leaf tissues. To mitigate this threat, plants have developed sophisticated signaling networks conferring so-called stomatal immunity (Arnaud and Hwang, 2015; Murata et al., 2015). Guard cells recognize various PAMPs, resulting in PAMP-triggered stomatal closure through the activation of downstream signaling components (**Figure 2A**). However, according to a coevolutionary model between plants and their pathogens known as the zigzag model, some adapted pathogens have developed phytotoxins (e.g., coronatine and syringolin A) and effectors (e.g., avirulence protein B [AvrB], hrp-dependent outer protein F2 [HopF2], HopM1, HopX1, and HopZ1) to overcome stomatal immunity and use open stomata as their entry point into the leaf apoplast space (Melotto et al., 2017). Recently, Lie et al. also revealed that *Xanthomonas oryzae* pv. *oryzicola* (*Xoc*) secretes the bacterial effector AvrRxo1 to impair stomatal immunity by inducing the degradation of rice PYRIDOXAL PHOSPHATE SYNTHASE 1 (OsPDX1) involved in ABA biosynthesis (Liu et al., 2022a). Mechanisms of immunity by stomatal closure and their relationship with humidity have been covered in previous reviews (Melotto et al., 2017; Aung et al., 2018). Notably, after pathogens invade internal plant tissues, stomatal closure can support conditions of apoplast hydration auspicious for pathogen colonization. Therefore, we focus here on the most recent mechanisms regulating stomatal conductance after pathogen entry.

Since water is essential for the survival of pathogens as well as plants, pathogens have to work hard to obtain water when inside their host plants (Beattie, 2016). Water soaking is a common disease symptom visible as leaf spots caused by virulent bacterial pathogens (Davis et al., 1991; Reimers and Leach, 1991). Bacterial pathogens (e.g., *Pst* DC3000) induce water soaking to establish a favorable colonization milieu by using their effectors (e.g., WtsE, AvrHah1, HopM1, and AvrE1) (Ham et al., 2006; Schornack et al., 2008; Xin et al., 2016). For instance, Xin et al. identified two effectors (HopM1 and AvrE1) that induce water soaking in Arabidopsis and demonstrated the molecular mechanism by which HopM1 promotes apoplast hydration for bacterial proliferation (Xin et al., 2016). Arabidopsis HopM1 INTERACTOR 7 (AtMIN7), which is an ADP ribosylation factor–guanine nucleotide exchange factor (ARF-GEF) localized to the *trans*-Golgi-network/early endosome and involved in vesicle trafficking, is identified as a binding partner of HopM1 during a yeast two-hybrid (Y2H) screen and confirmed by pull-down assay (Nomura et al., 2006). AtMIN7 contributes to PTI and ETI, and the *Pst* DC3000 effector HopM1 induces its degradation through the host 26S proteasome to suppress plant innate immunity (Nomura et al., 2011). Since AtMIN7 also plays a critical role in limiting fluid loss from plant cells, HopM1-mediated AtMIN7 degradation results in apoplast hydration and provides the favorable water condition needed for *Pst* DC3000 colonization; notably, high ambient humidity is required for water soaking (Beattie, 2016; Xin et al., 2016). Moreover, HopM1 and AvrE1 increase the expression of ABA-associated genes through transcriptome reprogramming and by raising ABA contents in guard cells (Roussin-Léveillée et al., 2022). The guard cell–specific ABA transporter ABCG40 is necessary for HopM1-mediated water soaking (Roussin-Léveillée et al., 2022), while AvrE1 activates ABA signaling through the inhibition of type one protein phosphatases (TOPPs), thereby suppressing SnRK2s (Hu et al., 2022). Therefore, *Pst* DC3000 utilizes HopM1 and AvrE1 to activate ABA signaling, inducing stomatal closure for water soaking after having invaded the plant inner space.

To prevent water soaking, plants promote stomatal reopening to establish a drier apoplast environment in pathogen-infected cells (**Figure 2B**). In rice, the *osaba1* mutant provided genetic evidence that increased stomatal conductance can enhance disease resistance to *Xoo* (Zhang et al., 2019). OsWRKY114 negatively regulated stomatal closure and conferred innate immunity against *Xoo* by repressing ABA signaling (Son et al., 2022; Song et al., 2022). Finally, in Arabidopsis, Lie et al. elucidated the molecular mechanism of stomatal immunity by which stomata reopen following effector-triggered stomatal closure (Liu et al., 2022b). They identified a class of small peptides, named the SMALL PHYTOCYTOKINES REGULATING DEFENSE AND WATER LOSS (SCREWs), and their receptor, the PLANT SCREW UNRESPONSIVE RECEPTOR (NUT), a member of the LRR-RK family. Flg22 treatment increases the expression of *SCREW*s and *NUT*, and recognition of SCREWs by NUT promotes the heterodimerization of NUT with BAK1. The NUT/BAK1 complex phosphorylates and enhances the phosphatase activity of ABI1 and ABI2, thus inhibiting the OST1/SnRK2.6-SLAC1 module whose activity promotes stomatal closure. As a result, plants can increase stomatal conductance to prevent water soaking through apoplast dehydration.

## The effects of carbon dioxide levels on stomatal immunity

Since the industrial revolution in the second half of the 18^th^ century, the concentration of atmospheric CO_2_ has begun to increase at an alarming rate. The Mauna Loa Observatory forecasts that the 2022 annual average CO_2_ concentration will be 418.3 ± 0.5 parts per million (ppm). This trend is expected to continue and reach 730–1000 ppm by the end of the 21^st^ century (Alley et al., 2007). Elevated CO_2_ levels can increase the yield of C_3_ plants by enhancing photosynthesis, but will not benefit C_4_ plants (Long et al., 2006). High CO_2_ levels will also affect plant–pathogen interactions. However, the effects of CO_2_ concentrations on plant defense mechanisms depend on specific plant–pathogen interactions and are complex (Noctor and Mhamdi, 2017). Moreover, the detailed underlying molecular mechanisms are not yet well known. Therefore, we provide below an overview of the best-documented effects of high CO_2_ on plant defense mechanisms related to stomata and photorespiration.

Like humidity, atmospheric CO_2_ concentrations control stomatal immunity. CO_2_ promotes stomatal closure through complex signaling networks (Zhang et al., 2018). First, atmospheric CO_2_ enters guard cells *via* the PLASMA MEMBRANE INTRINSIC PROTEIN (PIP) aquaporins, followed by the conversion of CO_2_ to bicarbonate (HCO_3_^−^) by beta carbonic anhydrases (βCAs) to activate downstream signaling events. Indeed, several studies have shown that the ubiquitous βCA enzymes are involved in the plant defense response. In Arabidopsis, genetic evidence demonstrated that βCA1 and βCA4 contribute to CO_2_-induced stomatal closure by converting CO_2_ into HCO_3_^−^ (Hu et al., 2010). The CA activity of βCA1 is required for a full defense response against avirulent *Pst* DC3000 carrying the effector AvrB (Wang et al., 2009). In addition, the quintuple mutant *βca1 βca2 βca3 βca4 βca6* exhibited a partial reduction in SA sensitivity (Medina-Puche et al., 2017). However, Zhou et al. showed that, despite impaired stomatal closure preventing pathogen entry, PTI-mediated SA-dependent immunity against virulent *P. syringae* was enhanced in the *βca1 βca4* double mutant (Zhou et al., 2020). Furthermore, they revealed that the PRR-mediated downregulation of *βCA1* and *βCA4* expression was attenuated by high CO_2_. These results suggest that CO_2_ concentration and βCAs regulate plant immunity positively or negatively as a function of compatible and incompatible interactions with the incoming pathogen. In tobacco (*N*. *tabacum*), βCA SA-BINDING PROTEIN 2 (SABP2) exhibits lipase activity and confers SA-dependent immunity against tomato mosaic virus (Kumar and Klessig, 2003). Similarly, SABP3 has antioxidant activity and confers HR triggered by Pto-mediated recognition of the effector AvrPto (Slaymaker et al., 2002). In addition, silencing of *SABP3* increases susceptibility to *Phytophthora infestans* (Restrepo et al., 2005). The expression of *CA* (accession number BQ113997) increased in potato (*Solanum tuberosum*) inoculated with an incompatible *P. infestans* strain, while it was downregulated in potato inoculated with a compatible *P*. *infestans* strain. Recently, Hu et al. also showed that βCA3 confers plant basal immunity in tomato (Hu et al., 2021b). High CO_2_ and *Pst* DC3000 increases the induction of *βCA3* expression by the transcription factor NAC43, while the phosphorylation of the serine 207 residue of βCA3 by GRACE1 (GERMINATION REPRESSION AND CELL EXPANSION RECEPTOR-LIKE KINASE 1) results in the activation of plant basal immunity related to the cell wall regardless of stomatal movement or SA signaling.

After converting CO_2_ into HCO_3_^−^, ABA signaling has a central role downstream of the convergence point of CO_2_ for stomatal closure (Webb and Hetherington, 1997; Negi et al., 2008). Dittrich et al. argued that PYL4 and PYL5 are essential for CO_2_-induced stomatal closure in Arabidopsis (Dittrich et al., 2019). However, CO_2_-induced stomatal closure appears to be triggered by an ABA-independent pathway downstream of OST1/SnRK2.6 without direct activation of OST1/SnRK2.6 (Hsu et al., 2018). Another group also reported results in support of this idea. They developed a SnRK2 activity sensor called SNACS based on Förster resonance energy transfer (FRET) and showed that, although basal ABA levels and SnRK2 signaling are essential for CO_2_-induced stomatal closure, CO_2_ signaling did not activate SnRK2s including OST1/SnRK2.6 and PYL4 and PYL5 were also not required (Zhang et al., 2020). Therefore, it remains controversial whether CO_2_ signaling can act upstream of SnRK2 in the ABA signaling cascade.

Moreover, recent studies indicated that ROS signaling is also important for CO_2_ signaling for stomatal closure. In Arabidopsis, ROS signals as well as ABA signals are necessary for CO_2_-induced stomatal closure (Chater et al., 2015). He et al. showed that ROS produced by both cell wall peroxidases and NADPH oxidases, together with phytohormones (SA, JA, and ABA), play an important role in CO_2_-signaling during stomatal closure (He et al., 2020). However, the detailed molecular mechanisms by which ROS modulate CO_2_ signaling are still unknown. Therefore, we discuss below the effects of CO_2_ on ROS generation and plant immunity.

## The effects of carbon dioxide on peroxisome-derived hydrogen peroxide

Photorespiration was once considered as a wasteful process because it is inefficient compared to the Calvin cycle and occurs when photosynthesis cannot operate. However, many studies have since shown photorespiration is involved in and required for various plant processes (Shi and Bloom, 2021). In particular, photorespiration has a crucial role in plant defenses due to ROS generation (Sørhagen et al., 2013). Hydrogen peroxide (H_2_O_2_) is a non-radical ROS that is deeply associated with plant defense responses (Smirnoff and Arnaud, 2019). It is produced mainly in leaf peroxisomes during photorespiration, with peroxisomal glycolate oxidase (GOX) and catalase (CAT) acting as major positive and negative regulators of its production, respectively (Foyer et al., 2009; Corpas et al., 2020).

Photorespiration and the Calvin cycle are competitively controlled by ribulose-1,5-bisphosphate carboxylase/oxygenase (Rubisco); thus, high CO_2_ levels decrease photorespiration (Long et al., 2004; Busch, 2020). Therefore, high CO_2_ would be expected to repress plant immunity. However, several studies have shown that high CO_2_ can increase plant defense responses including SA and JA (Noctor and Mhamdi, 2017). In addition, CAT2 was shown to be involved in SA-mediated auxin and JA inhibition of resistance against biotrophs (Yuan et al., 2017). Recently, Williams et al. demonstrated that CO_2_ influenced resistance to biotrophic and necrotrophic pathogens differently in Arabidopsis (Williams et al., 2018). Under high CO_2_ conditions (1200 ppm), resistance to both the biotrophic oomycete *Hyaloperonospora arabidopsidis* and the necrotrophic fungus *Plectosphaerella cucumerina* increased compared to ambient CO_2_ (400 ppm). SA appeared to play a minor role in resistance to the biotrophic pathogen, while JA conferred strong resistance against the necrotrophic pathogen. At low CO_2_ (200 ppm), resistance to *H*. *arabidopsidis* was enhanced through photorespiration-derived H_2_O_2_ production, whereas resistance to *P*. *cucumerina* declined.

## Prospects of genome editing for climate resilient crop development

Advances in biotechnology have opened up the possibility of overcoming the deleterious effects of climate change on crop plants. Induction of plant innate immunity compromised by climate change improves disease resistance to pathogen under the unfavorable environmental condition, but the constitutive activation of plant immune response retards growth and reduces crop productivity. To address this problem, scientists focused on the strategy to activate plant defense response spatiotemporally using pathogen-induced promoters and pathogen-responsive upstream open reading frames (Kim et al., 2021). However, this method cannot be free from the issue of genetically modified organisms. Therefore, the genome editing technologies based on SDNs (e.g., CRISPR/Cas9) are necessary for the development of climate resilient crops. However, even though genome editing has successfully increased the disease resistance of various crops, there are still significant hurdle to its application to climate change adaptive crop development due to the negative effects of mutations on the crop’s performance (Karavolias et al., 2021). Therefore, in order to cope with the future food resource crisis, understanding the various plant immune mechanisms affected by climate change and identifying elite genes that can improve disease resistance through genome editing will be one of the most efficient ways to develop climate resilient crops.

## Conclusion

We are currently living in an unprecedented era of climate change. The consequences of this changing climate may diminish crop production and access to nutrients for all living creatures, concomitantly with the faster adaptation of microorganisms including phytopathogens due to their short life cycle and rapid propagation compared to other and more complex species, causing more severe damage to crop plants. It is clear that the damage to global crop security due to biotic stresses will pose a great challenge to human life in the future. Scientists have recently achieved remarkable progress in this field. Here, we provide an overview of the known and anticipated effects of climate change such as temperature, high humidity, and CO_2_ on plant immunity mechanisms. The current efforts to understand how climate change will impact plant immune systems and to develop more efficient NPBTs will make it possible to overcome the incoming crisis through crop improvement that can minimize damage and preserve yields in future pathogen-friendly environmental conditions.

## Author contributions

SS conceptualized and wrote the manuscript. SRP supervised. All authors contributed to the article and approved the submitted manuscript.

## Funding

This research was funded by Research Program for Agricultural Science and Technology Development (Project No. PJ01661001), and supported by the 2022 Fellowship Program (Project No. PJ01661001) of the National Institute of Agricultural Sciences, Rural Development Administration, Republic of Korea.

## Conflict of interest

The authors declare that the research was conducted in the absence of any commercial or financial relationships that could be construed as a potential conflict of interest.

## Publisher’s note

All claims expressed in this article are solely those of the authors and do not necessarily represent those of their affiliated organizations, or those of the publisher, the editors and the reviewers. Any product that may be evaluated in this article, or claim that may be made by its manufacturer, is not guaranteed or endorsed by the publisher.
